# The Therapeutics of Asiatic Cholera

**Published:** 1893-02

**Authors:** I. E. Atkinson

**Affiliations:** Professor of Materia Medica and Therapeutics, University of Maryland


### THE THERAPEUTICS OF ASIATIC CHOLERA.*

BY I. E. ATKINSON, M. D.,

Professor of Materia Medica and Therapeutics, University of Maryland.

It must have occurred to most of us, in listening to the very succinct and exhaustive statements of Dr. Lattimer regarding the geographical distribution of this great modern scourge of our race, with a good deal of humiliation, that the art of which we are practitioners has had very little to do in bringing about the subsidence of these various epidemics and pandemics. The epidemics seem to have spread through most countries at their own sweet will, and to have subsided for reasons which are largely beyond our powers of recognition. But while we have never had a remedy that has a definite antagonistic influence over the progress of this disease, a careful observation of the laws of hygiene places a community fairly beyond the reach of its ravages. Undoubtedly, as we all know, the number of vaunted specifics for the treatment of cholera is more than we care to consider to-night. After a brief experience with these remedies, the results of which were phenomenal in the hands of certain observers, the rose-colored reports that were given have failed to be confirmed. I will not attempt to enumerate the various specific remedies, so-called, that have been brought forward from time to time, but will begin my subject, basing my remarks upon knowledge of the disease, made more definite by the discovery of the comma bacillus by Koch.

This discovery, while it has placed in our hands as yet no specific treatment, has given us very definite ideas of the difficulties we have to contend with and the indications for treatment and for the administration of remedies, if we be so fortunate as to

*Read before the Clinical Society of Maryland, November 4, 1892.

find them. To enumerate seriatim the indications for the treatment of cholera : Our first object is the limitation of the development of the organism that is the cause of the disease ; and this therapeusis is directed to the alimentary canal. The second indication is the neutralization of the poison that is produced, by the vital activity of these organisms, in the intestinal canal. The third indication is the elimination as early as possible of the poison from the blood into which it has gained access from the intestines. The fourth indication is the restoration of the blood to its normal condition ; that is, to its proper degree of fluidity. It is very easy to state these therapeutic indications, but it is far more difficult to supply the therapeutic measures. Still, with the knowledge we have of the nature and the operation of the disease and the conclusions we draw with regard to the poison which the organisms produce, we are infinitely more favorably situated for struggling with the disease than we have ever been before.

In the space which I have a right to occupy, I can only say a few words in regard to the prophylaxis of cholera. That, however, is the most important aspect of the case. I shall begin with the bedroom of the patient, his house, his surroundings and the disposal of excreta. Most of this I can dispose of in a very few words, because of our knowledge of antisepsis and the influence of minute organisms in the production of disease, which is the alphabet of prophylaxis. Still, it may be proper to speak a little in regard to the exact procedures that should be followed out in case of cholera in the household. The attendants of the patient should be limited. The bed should be prepared in such a way that no permanent contamination of the bedding can take place. For this purpose a waterproof blanket should be spread upon the bed and another upon the floor under it, that discharges shall not soil the carpet or flooring. The attendants should be clad in waterproof clothing. Antiseptic liquids should be at hand, and there should be a constant washing of hands. Attendants should be strictly instructed not to convey their hands to their mouths and not to feed themselves with their hands. They should use mouth washes and antiseptic solutions of weak strength snuffed into the nose. With regard to the water and food supply I should speak more definitely. Of course every one knows that the drinking water should never be used unless it has been thoroughly and recently boiled. In passing, I should say that I do not regard our Baltimore water supply as at all beyond suspicion. From my personal knowledge of the condition of Lake Roland and of its affluents, and the amount of sewage and drainage that goes into it, I believe that under proper conditions it may be a source of danger. The Gunpowder water supply I regard as being as nearly perfect as can be had. The water should be absolutely boiled. The food of the patient should be freshly prepared and cooked. Uncooked fruits of all kinds should be avoided. Milk should be especially avoided unless recently boiled in a sterilizer. The food should be of a plain, nutritious character, but it should not be long free from the necessary amount of heat to destroy the activity of the bacillus.

Passing a little more into the specific conditions, let me say that experience seems to show that a healthy digestive apparatus is a fairly good guarantee against any attack of cholera. Those persons suffering from organic gastric derangements are the persons who are most prone to develop the symptoms of cholera so far as we know. It is claimed, I know not with how much truth, that the natural healthy acid secretion of the stomach is inimical to the development of the bacillus. It goes without saying that the excreta should be properly disinfected. For this purpose various agents are recommended. An ordinary five per cent. solution of carbolic acid seems to be most popular, and we are advised that the excreta should be allowed to remain in this solution for some time before thrown out. Ordinary commercial sulphuric acid, three or four drachms to be added to each stool, is recommended. The various corrosive sublimate solutions can be employed.

Suppose the precautionary measures have been unavailing and we have the patient in the condition of preliminary diarrhœa of cholera. This is an uncertain condition. Unquestionably in cholera epidemics diarrhœas are very prevalent. Often they are of no consequence; often after a time they develop into true cholera. The outcome we cannot always foretell, and we should carefully treat the diarrhœa. Nearly all recommendations for treatment of this preliminary diarrhœa include opium. This seems to have been a universal practice and unquestionably it is an excellent practice in the ordinary diarrhœas which may be choleratic ; but whether this plan of treatment is efficient in true choleratic diarrhœas is a point upon which I am not prepared to speak with conviction. Certain it is that nearly all observers at the present time condemn opium in the treatment of cholera; not hesitating to use it to relieve extreme pain, but as a remedy for the treatment of cholera itself it is condemned. I read an article yesterday evening by Sir George Johnson, in which he observed that in some of Koch's experiments he was not able to produce cholera in rabbits experimentally until he had first given them opiates. At all events whether the use of opiate preparations in the preliminary diarrhœa of cholera is to be recommended or not, there seems to be almost a universal sentiment amongst recent observers that opium is not the proper remedy to employ in the developed disorder. Many authorities recommend that the preliminary diarrhœa of cholera should be treated by purgatives and that if opium is given in these cases it should always be in association with a purgative, and experience seems to show that castor oil is the most efficient of these. The castor oil is given with more or less persistence during the treatment of the diarrhœa. Of course the patient should be kept in bed until the diarrhœa is cured.

The use of acids has been very much recommended in these preliminary conditions. It is claimed that the acid condition of the stomach is inimical to the development of cholera and it has seemed that this antagonistic condition of the stomach might be increased by giving the proper acid; therefore small doses of hydrocholoric acid and other mineral acids are given and with a fair degree of justification. But these are merely accessory means for combating the choleratic diarrhœa in the initial stage.

I approach now a drug that has recently been recommended as a specific for cholera ; and certainly if we could rely upon the statements (and we all know how unreliable statements made from a few observations are) we well might suppose that in this remedy we have one that has a specific influence in antagonizing the poison. This drug is salol. Lowenthal has written extolling its use. Gonzales lost only three patients in fifty-three cases of cholera treated with salol. Nicholson treated thirteen cases; all recovered. Hehir treated eighteen cases with corrosive sublimate with a mortality of 44.7 per cent.; eleven with salol without a death. If the list went on as this starts we would have every hope of having a specific in cholera as we have a specific in malarial fever or syphilis or acute rheumatism; but recent reports, especially from the hospitals of Hamburg, during the epidemic, from a number of physicians, declare that salol gave no good results whatever in their hands. While at the present time we are not prepared to deny a specific influence to salol we are not prepared to accept it as a specific or remedy that exerts a pronounced influence over the course of the disease. It undoubtedly has a disinfecting influence on the intestinal canal, and I am inclined to think that I will use it if I have to treat cases of cholera.

The causes of death in cholera seem to be due, first of all to the loss of fluid from the body, and in the second place to the chemical substance generated by the bacillus, which acts as a poison on our bodies. Now, the effort to meet this indication has called forth certain novel methods of treatment which seem to promise a great deal. The practice of what is called "enteroclysis" it is claimed gives marked results in the treatment of cholera. This practice has its greatest advocate in Cantani, of Naples, although he was not the first to practice it. It has received its highest praise from him, and its most extensive application has come from his description of its use in this disease. He claims that under this enteroclysis and the method of an injection of a saline fluid under the skin—hypodermoclysis—to have had 70 per cent. of recoveries. What is enteroclysis ? We are told that in the very early stages of cholera there should be injected into the rectum a fluid containing tannic acid, because this acid exerts an astringent influence on the mucous membrane of the bowel and also has a destructive action upon the vitality of the bacillus. From five to twenty grammes should be used to the litre of water or about 75 to 300 grains to the quart of water, at a temperature of two or three degrees above the normal temperature of the body. The injection is allowed to run in slowly and is frequently repeated. This succeeds in washing out to a certain extent the large intestine, and Cantani claims—and there I think we will be disposed to question the accuracy of his claims—that the fluid goes beyond the ileo-cæcal valve. I doubt whether the liquid can be made to reach beyond the ileo-cæcal valve, or if so, then only in very small quantities. This doubt cannot but make us feel some hesitation as to the accuracy of other statements. At first this solution of tannin was made in infusion of chamomile. I do not think that any one can claim any specific virtues for the chamomile, and it seems to me that inasmuch as in the cholera we should lay aside everything that will embarrass our action, then, unless chamomile has a therapeutic value, we should dispense with it. Then, too, a certain amount of tincture of opium, 30 to 50 drops, may be added to the injection. But so far as I can discover, the essential agent is a solution of tannin in warm water. As the algid stage comes on, and the patient begins to lose, by vomiting and purging, enormous quantities of water and fluid characteristic of the disease, this enteroclysis is supplemented by hypodermoclysis ; and that consists in introducing under the skin a solution of common salt, the normal salt solution of about 75 per cent., or one may use a teaspoonful in a quart of water. According to Cantani's recommendation it is combined with carbonate of sodium. One may use a drachm of chloride of sodium and forty-five grains of carbonate of sodium to a quart of water at thirty-nine degrees centigrade. This is introduced by means of a canula into the subcutaneous tissue, and usually in the ileo-costal region. The canula is introduced, the fluid is allowed to run in under gentle pressure through tubes and vessels that have been carefully antisepsitized. The pressure is so gentle that it will take from thirty to fifty minutes to run in. Instead of having only one point, it is desirable to divide the fluid into equal quantities and run it into different parts of the body. This forms tumors which gradually disappear. This method, it is claimed, and especially in the Hamburg hospitals during the last epidemic, has done marvels and has brought people right up from the very jaws of death. But further observations among those who practiced among the cholera patients in Hamburg seem to place the intravenous injection of this fluid above the hypodermoclysis. Quite a number of physicians have concluded that intravenous injections produced results marvelously greater than those of the hypodermic injections. I remember a statement that one physician made, that where a patient had been brought out of a state of collapse by the intravenous injection of the saline fluid, and falling again into collapse, the hypodermic injection did not succeed in restoring him as the intravenous injection did, while the intravenous injection again and again would bring the patient out of this condition. It seems from a purely theoretical point of view that those intravenous injections should not produce results so marvelously better than the other. By this method one runs the risk of thrombosis and of introducing other substances into the veins. Such results, however, did not occur in Hamburg. If there is any power of absorption in the connective tissues at all, and if it can be shown that this fluid is taken up, it would seem reasonable that there should be this marvelous difference. In the discussion a number of other speakers preferred intravenous injection. When the algid state seems to be past and the patient passes into the typhoid state of cholera, it is recommended that enteroclysis be again resorted to, not with tannic solutions, but with saline solutions for the purpose of restoring the blood to its proper condition. These solutions may begin with from two to five grammes to the litre or one to two drachms to the quart, and they may be increased until the proportion of salt in the solution may approach fifteen or twenty per cent. This should be used along with the hypodermoclysis or intravenous injection of the saline fluid. Recently arterial injection has been recommended, but I have not been able to find any reliable data about it, and therefore I only refer to it.

This constitutes what seems to be the most promising treatment of Asiatic cholera, and nearly every one who has practiced it speaks of it in terms of praise.

I want to refer again to the point that opium not only seems not to have a beneficial, but to have a positively meretricious influence over the course of Asiatic cholera. I wish to refer again to the statement that the results in the treatment of cholera in earlier years when calomel and castor oil were used appear to have been better than they were under the treatment by opium. Certainly the results of the treatment of cholera in earlier years by calomel and castor oil appear to have been better than they were under the treatment by opium. Certainly the treatment of cholera by opium and by the various mineral and vegetable astringents does not give a percentage of cures that in any degree encourages us to persevere in their use. Here we have a method that is under trial. It is certainly worth careful investigation and I am sure that if I were called upon to treat cases of cholera to-morrow, I should at once put this method of treatment into practice.

